# Catching a floating thrombus; a case report on the treatment of a large thrombus in the ascending aorta

**DOI:** 10.1186/s13019-017-0600-x

**Published:** 2017-05-19

**Authors:** Gijs Eduard de Maat, Giorgio Vigano, Massimo Alessandro Mariani, Ehsan Natour

**Affiliations:** Department of Cardiothoracic Surgery, University of Groningen, University Medical Center Groningen, P.O. Box 30.001, 9700 RB Groningen, The Netherlands

**Keywords:** Aortic disease, Ascending aorta, Floating thrombus, Case report

## Abstract

**Background:**

The ascending aorta is an uncommon site for non-infective thrombus. In non-aneurysmal and non-atherosclerotic vessels this condition becomes extremely rare, while it represents a source of potential cerebral and peripheral embolic events. Currently, there is no consensus in the guidelines on how to treat a free floating thrombus in ascending aorta, therefore we present our decision making process and therapeutic strategy.

**Case presentation:**

A healthy 48-year-old man was hospital admitted with acute abdominal pain. CT-scan showed a right renal embolism in presence of a defect in the distal ascending aorta suggestive for thrombus. After heart team discussion the patient was scheduled for surgery and successfully underwent an emergent thrombus removal. Also, owing to multiple aortic wall insertions, the ascending aorta was replaced. The patient’s recovery was uneventful and histological examination showed no signs of connective tissue disorders of aortic wall while confirmed the thrombotic nature of the mass.

**Conclusions:**

We present a patient with a floating thrombus in the ascending aorta who underwent an ascending aorta replacement. While angio-CT scan led to a prompt diagnosis, intraoperative epi-aortic echocardiography allowed to define precise location of thrombus, minimizing operative risk. This case demonstrates that multi-disciplinary heart team discussion is essential to define a successful strategy, that surgical treatment is feasible with specific tools such as epi-aortic echocardiography.

## Background

The ascending aorta is an uncommon site for non-infective thrombus. In non-aneurysmal and non-atherosclerotic vessels this condition becomes extremely rare, while it represents a source of potential cerebral and peripheral embolic events. The exact mechanism of thrombogenesis is still unknown although several speculations have been proposed. How to treat a free floating thrombus in ascending aorta is still debated and no consensus exists yet [1–4]. We report and discuss the surgical management of a free large floating thrombus in the distal ascending aorta, which already had partially embolized to the right kidney. Owing to its rarity and to the lack of a general agreement heart team discussion played a fundamental role in the decision making process. Since concerns regarding indication and efficacy of antiplatelets are still present, conventional replacement of the weak portion of aorta could represent a valid and effective treatment reducing recurrence rate of thrombi.

## Case presentation

**Fig. 1:**
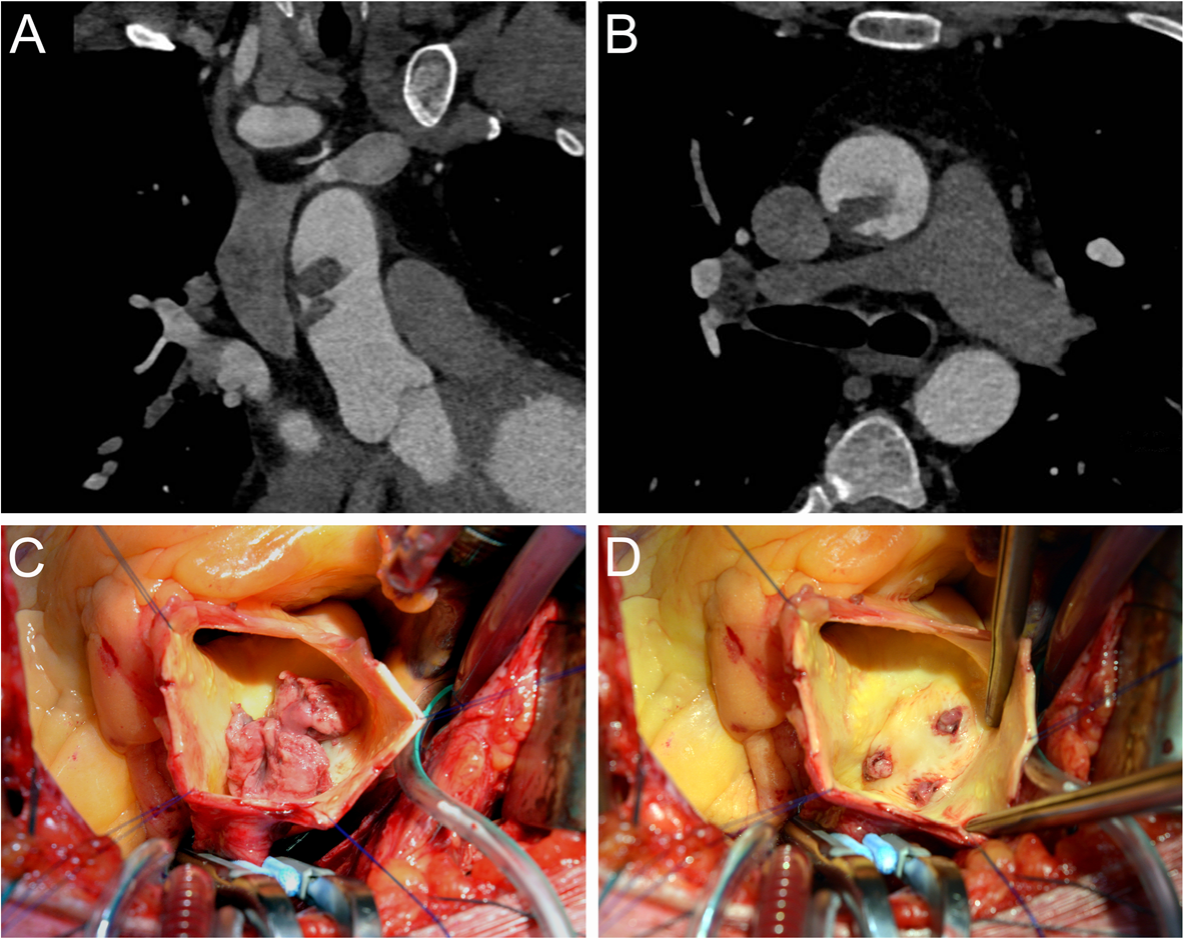
**a-b** Pre-operative computed tomography of the aorta showed a large structure in the ascending aorta. **c-d** Intra operative images of the large thrombus in the ascending aorta and the atherosclerotic insertion in the aortic wall

A 48-year-old male with unremarkable medical history was hospital admitted owing to acute abdominal pain. Computed tomography (CT) scan of the abdomen showed an acute right renal embolism in presence of thrombotic emboli in the distal superior mesenteric artery. Further investigation by means of a thoracic angio-CT scan showed a large intra-luminal free structure in the ascending aorta suggestive for thrombus (Fig. 1a and b) with no signs of aortic dissection or intramural hematoma. During multidisciplinary heart-team discussion all possible strategies were explored. According to a high embolism risk conservative medical treatment by heparinization immediately appeared inappropriate and the patient was scheduled for emergency surgical thrombus removal. The calculated Logistic Euroscore II was 1.31%. The operation was performed through a median sternotomy, on cardiopulmonary bypass. Once the pericardium was opened, extreme care was put in manipulating aorta. Epiaortic echocardiography was performed to precisely define the position of thrombus allowing an easy and safe cannulation of the proximal aortic arch. After aortic cross clamping, the ascending aorta was opened and selective cardioplegia was administered. A large thrombus was identified (Fig. 1c) located on a small atherosclerotic posterior-lateral plaque. After thrombus removal, a three-sites aortic wall insertion was observed (Fig. 1d). On account of these findings it was decided to replace the supracoronary ascending aorta, interposing a 30 mm Dacron vascular prosthesis conduit (Gelweave, Vascutek, Terumo, Inchinnan, UK). Neither deep hypothermic circulatory arrest nor cerebral perfusion was required. The patient fully recovered after an uneventful postoperative course and was discharged from hospital on 3 months vitamin K antagonist, followed by lifelong Aspirin. Histological examination of the aorta showed no signs of connective tissue disease while confirmed the thrombotic nature of the structure.

## Discussion

The evidence on the topic of floating thrombi in ascending aorta is very limited and dispersed. According to the International Guidelines (2014 ESC Guidelines on the diagnosis and treatment of aortic diseases; Section 9 Atherosclerotic lesions of the aorta; Chapter 9.2 Mobile aortic thrombosis) consensus is based on experts’ opinion (level of evidence C) and every case becomes peculiar and must be discussed in a Heart Team [1]. Kalangos et al. put the accent on differential diagnosis which no longer represents a challenge thanks to ever more advanced diagnostic imaging [4]. Meanwhile the pathophysiological mechanism of thrombi still remains unclear, as patients frequently do not suffer from coagulopathies, immunological disorders or malignancies. In non-aneurysmatic, non-atherosclerotic vessels and in absence of a clear etiology Behcet’s disease can be suggested as a possible cause, particularly among countries around Mediterranean Sea, but this was not our case. According to operative findings of a three-sites aortic wall insertion, we decided to replace that portion of aortic wall. Although no real atheroma was found and excised aortic wall appeared normal on histological and immunological examinations, we strongly recommend replacement of the involved portion of aorta, in order to prevent recurrences. On the other hand this could increase operative risk, depending on location of thrombus particularly in proximity of aortic arch. While conservative medical treatment certainly represents a cornerstone of primary approach in asymptomatic patients, management becomes demanding in presence of symptoms or distal embolism. Embolisms (e.g. cerebral) could significantly limit the surgical options. A few cases have shown that a conservative approach with anticoagulants represents a viable option [5, 6]. However Moris et al. suggested the strategy should be chosen based on peculiar characteristics of thrombus like its location, mobility, morphology, persistence of symptoms under anticoagulants and high risk of recurrence [7]. While the dimensions of the thrombus are never considered as main criterion, the location of thrombus strongly affects the possible surgical strategies. Thrombus removal under circulatory arrest and deep hypothermia has been proposed [3] as well as with normothermic aortic cross clamping [4]. In our case, thrombus was located in the distal ascending aorta, with inherent concerns. Arterial peripheral cannulation (e.g. femoral artery or right subclavian artery) could represent a valid option, minimizing manipulation of the aorta. Live epi-aortic echocardiography enabled to define the exact position and dimensions of the thrombus. These echo findings added information essential to determine the safe cannulation site.

Our patient was discharged from hospital on vitamin K antagonist for three months owing to previous renal embolism. After this period Aspirin has been started lifelong. A six months follow-up CT-scan showed no signs of thrombi. Since secondary prophylaxis still represents a debating issue, we propose an effective and feasible solution to treat thrombi in ascending aorta. In a field where no precise indications are available, we aim to contribute to develop specific guidelines and flowcharts that help consultants in the decision making process, ensuring the best care for patients.

## Conclusions

We present a patient with a floating thrombus in the ascending aorta. While angio-CT-scan led to a prompt diagnosis, intraoperative epi-aortic echocardiography allowed to define precise location of thrombus, minimizing operative risk. Since the treatment of floating thrombi in the ascending aorta is still debated and very little evidence exists, we report the decision-making process and strategy. In this presented case, we would like to underline the importance of the multi-disciplinary heart team and also the usefulness of intraoperative epi-aortic echocardiography. Due to the high risk of embolization and relatively low risk of surgical intervention, the ascending aorta was replaced. This case demonstrates that surgical treatment of a floating aorta is feasible and urgent intervention may offer a safe solution.

## Abbreviations

CT-scan: Computed tomography scan
